# Assessing Animal Welfare and Service Needs in a Community-Based Program

**DOI:** 10.3390/ani16162496

**Published:** 2026-08-11

**Authors:** Emily Patterson-Kane

**Affiliations:** ASPCA Research, New York, NY 10128, USA; emily.patterson-kane@aspca.org

**Keywords:** animal welfare, community outreach, needs assessment, urban populations

## Abstract

The American Society for the Prevention of Cruelty to Animals (ASPCA) Community Engagement (CE) program works with communities in New York City to improve animal welfare through accessible services and outreach to pet owners. To systematically identify welfare needs, the CE team developed a 10-item Animal Needs Assessment (ANA) tool and applied it to 519 cases (records that track from first report to resolution or loss of contact with a client) between January 2021 and September 2024. The most frequent needs were lack of enrichment or resting places, illness, behavioral needs and the removal of hazards. Social service referrals were associated with more serious animal needs, highlighting the intersection of human and animal vulnerability. Services were successfully delivered in 93% of cases, and client openness at first contact predicted successful follow-through. These findings suggest that assessment tools such as the ANA may help guide service planning and inform strategies for improving community-based animal welfare initiatives.

## Simple Summary

The American Society for the Prevention of Cruelty to Animals (ASPCA) Community Engagement program provides direct assistance to pet owners in communities in New York City. To better understand and address animal welfare needs, a 10-item Animal Needs Assessment (ANA) tool was created. In an analysis of 519 cases, common needs identified through ANA were lack of enrichment or resting places, illness, behavioral needs and the removal of hazards. Cases referred by social service agencies often involved more serious animal concerns, underscoring the link between human and animal vulnerability. These results highlight the value of practical assessment tools for targeting services and improving outcomes in community-based programs.

## 1. Introduction

Many pet owners face challenges in caring for their animals due to financial limitations, health issues, disabilities, lack of transportation, or limited access to essential supplies [1]. Access to care is an issue not only for veterinary care but also for obtaining other resources such as pet food [2], animal training [3], and grooming services [4]. To improve accessibility, some animal welfare agencies have implemented community-based outreach programs, bringing services for animals directly to homes, businesses, and local events. This approach has been effective for delivering veterinary care, promoting pet retention, and providing humane education [5,6,7].

The ASPCA Community Engagement (CE) program, which serves pet owners in New York City, aims to improve animal welfare and human well-being by keeping pets and people together whenever possible [8]. The CE team offers assistance with essential supplies (e.g., pet food, leashes, and crates), veterinary care, grooming, and other necessary services. Clients are referred to the program by social service agencies, law enforcement, animal shelter partners, other ASPCA service programs, and concerned citizens. In addition to directly providing subsidized or no-cost services, the CE team also provides referrals to social service agencies.

According to the Veterinary Care Accessibility Project [9], veterinary care access across New York City ranges from “nearly inaccessible” (15/100 in Bronx County) to “generally accessible” (70/100 in Richmond County). Other common challenges identified by this project in New York City include language barriers, poverty, and limited availability of transportation, all of which can affect access to services for both humans and animals.

A previous retrospective study of CE cases involving cat hoarding found that many pet owners in these cases expressed interest in spay/neuter services or relinquishment but faced barriers to accessing them—suggesting that these difficulties may contribute to hoarding situations [10]. Additionally, a survey of clients receiving grooming services identified financial limitations, lack of supplies, and concerns about accidentally harming their pets as major barriers; a small but significant portion of respondents reported that outdated vaccinations prevented them from accessing grooming services [11].

Expanding availability and access to data from community-based agencies could provide deeper insights into the animal welfare challenges faced by pet owners, informing strategies to prevent or address these issues. Data collection can assist community engagement programs, for example by assessing barriers to care [12], demonstrating the impact of outreach programs [6], or elucidating the intersections between human and pet health (One Health) [13].

To further capture and categorize animal welfare challenges in its service area, refine measurement of program impact, and inform future interventions, CE developed a plan to supplement its existing approach to case documentation and tracking (narrative descriptions) by introducing quantitative coding of animals’ needs via a 10-item animal needs assessment (ANA).

## 2. Materials and Methods

This study includes data from the five boroughs of New York City collected between January 2021 and September 2024. Data were collected at three time points: at referral, in interaction with the client (phone call and home visit), and in a follow-up phone call to the client.

At referral, the referral source and initial free text case description were recorded by the CE staff member receiving the call, including the most salient factors from the referral relating to the client, animals, needs, and any recent changes in the household.

This information was verified and further detailed during a phone call to the client, where they were given the option to opt in to a home visit. During the home visit, the CE case lead finalized their observations and developed a service plan. Immediately after this visit, they filled out a CE Case Assessment Form, which included 29 prompts as shown in Appendix A and the 10-item Animal Needs Assessment shown in Table 1.

These 10 items were based on workshopping, a consensus of CE staff members’ experiences and estimates of the most common animal needs they encountered. The ANA evaluates five environmental factors (food, water, hazards, resting place, and enrichment) and five animal factors (illness, injury, grooming, body condition, and behavior) and categorizes levels of concern as none, mild, moderate or serious. Levels of concern accounted for both seriousness and urgency of the need, with mild concerns not requiring immediate intervention, moderate concerns requiring intervention within 1–2 weeks, and serious concerns requiring intervention within 1–2 days (see Appendix A).

The data set included all cases closed from the advent of using the animal needs assessment (ANA) January 2021 until September 2024. These cases were extracted from the case management database (Civicore CMS, Neon One) and exported as a CSV file. The exported file included 540 events, of which 519 were unique first-time cases. The CE program does not offer repeated services; however, a small number of second (20) or third (1) cases were found to involve the same client, as a result of another referral being made after the prior case was closed. These 21 records were removed due to their small number, being out of the intended scope of services, and to maintain independence of case records.

After extraction, data were reviewed and organized in Excel (Microsoft^®^ Excel^®^ for Microsoft 365 MSO, Version 2507). Ambiguous or missing entries were resolved using information from the 29 assessment prompts. Statistical analyses were conducted in Stata 17 (StataCorp LLC, College Station, TX, USA, 2021.). Descriptive statistics were calculated for referral sources, household characteristics, animal welfare concerns, and services provided. Associations between categorical variables were evaluated using Fisher’s exact tests, while differences in continuous variables were assessed using *t*-tests or analysis of variance (ANOVA), with Sidak-adjusted post hoc comparisons where appropriate.

To explore patterns in services offered, principal component analysis (PCA) was conducted on binary service indicators (0 = service not offered, 1 = service offered) using the correlation matrix. Components with eigenvalues greater than 1.0 (Kaiser criterion) were retained and interpreted according to their loading patterns.

To identify factors associated with successful completion of all recommended services, binary logistic regression was performed with service delivery outcome (all services received vs. not all services received) as the dependent variable. Candidate predictors were screened using bivariate analyses, and variables showing associations at *p* < 0.05 were entered into the multivariable model. Variables representing subsets of broader included measures (e.g., number of cats owned, total serious concerns) were excluded to reduce redundancy. Odds ratios, 95% confidence intervals, and *p*-values were reported.

Free-text referral descriptions were coded in QDA Miner 6 (Provalis Research, Montreal, QC, Canada, 2023). Codes were developed inductively from recurring themes in the referral narratives and grouped into categories relating to clients, animals, barriers to care, and presenting concerns. Co-occurrence among codes was visualized using a two-dimensional concept map, in which node size reflected coding frequency and distance between nodes reflected the tendency of codes to occur together in the same case.

## 3. Results

### 3.1. Referrals

**Figure 1:**
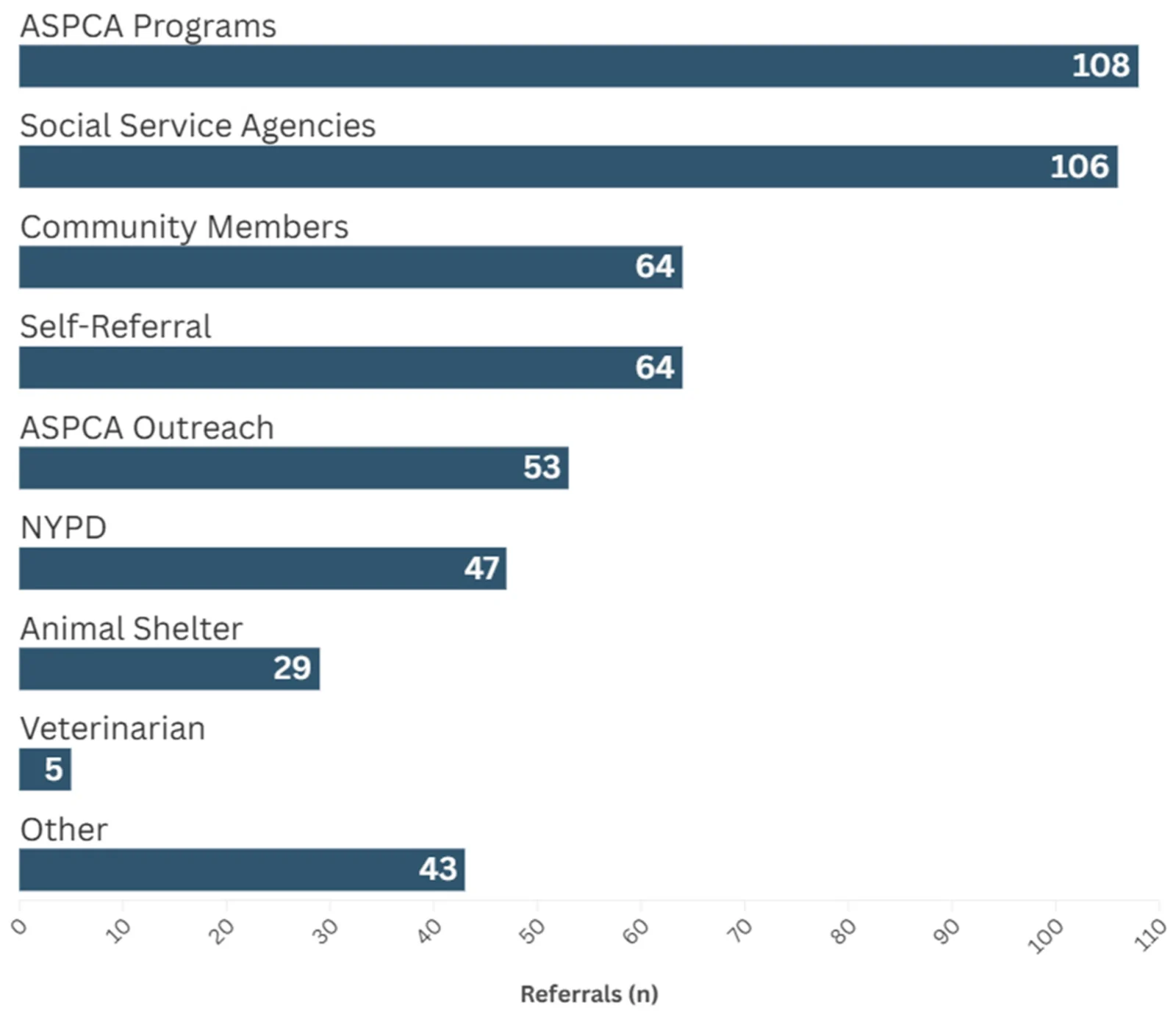
Sources for cases referred to the ASPCA Community Engagement team.

Referrals were received from various sources (Figure 1). The most common referrer types were other ASPCA programs (108/519, 21%) and social service agencies (106/519, 20%), which included all private or governmental organizations that assisted clients with housing, health, or other services important for their wellbeing. Referrals from social service agencies were associated with significantly higher numbers of serious animal needs (*M* = 1.25, *SD* = 0.20), compared to those from other referral sources (*M* = 0.77, *SD* = 1.55), t(517) = −2.61, *p* = 0.01.

**Figure 2:**
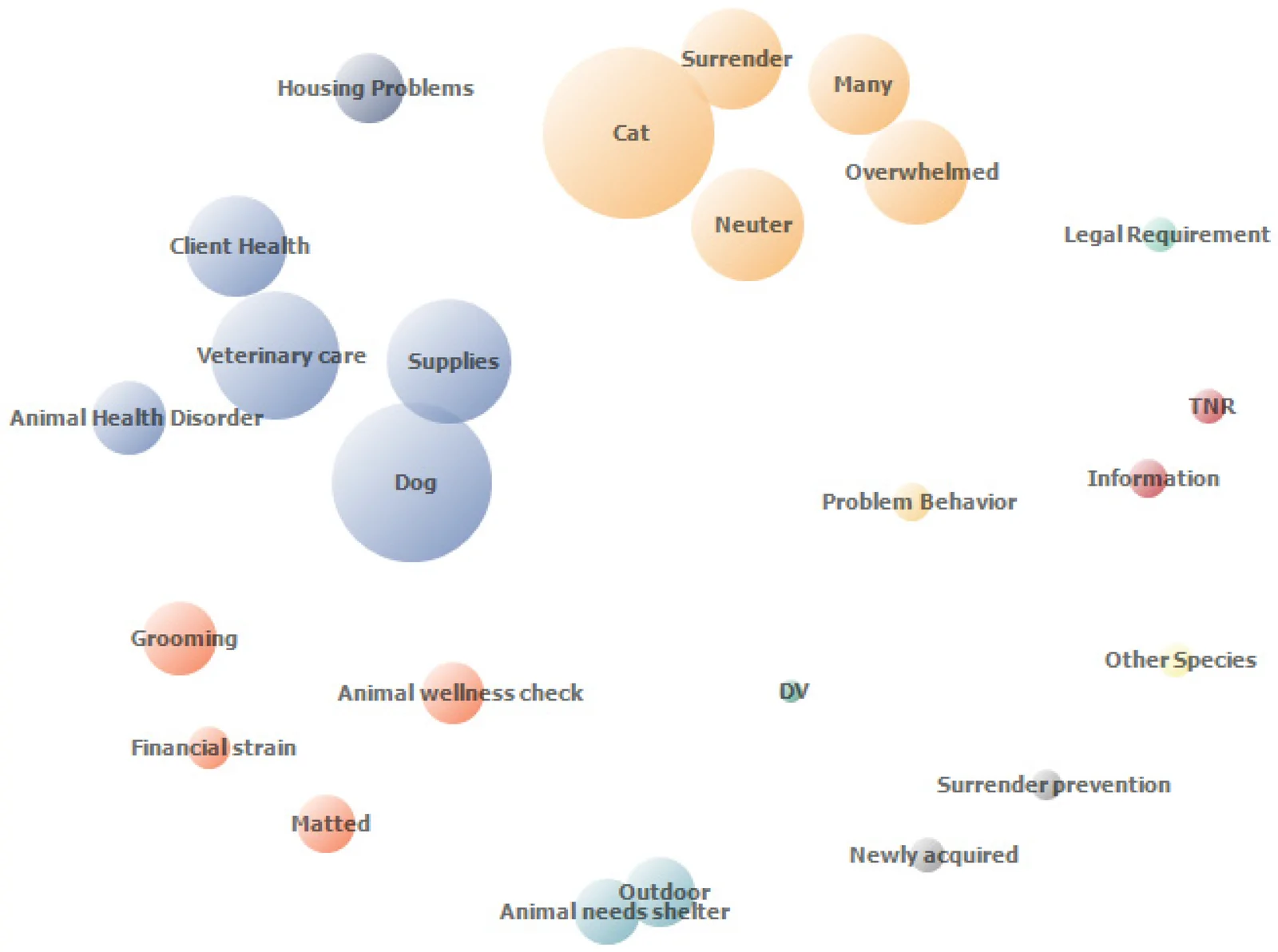
A map of factors mentioned in referrals to the ASPCA Community Engagement team.

The free text case description at referral was coded by the author according to identifiable barriers, clients, animals, and descriptors of difficulties being experienced by the person or animal. These codes were used to create a two-dimensional concept map of information mentioned in referrals, where the size of the circles reflects the frequency with which each code was applied and proximity of circles to one another reflects factors that tended to co-occur in the same case (Figure 2).

The map of referral factors indicated several noticeable clusters indicated by proximity and color, listed in descending order of size: clients affected by illness, disability, or age who needed veterinary care and supplies for dogs; clients with more cats than they were able to care for; clients requiring population control for animals in their care (spay/neuter, rehoming: “many” animals was defined as either a number of pets the client expressed was too many, or ten or more animals with welfare needs); clients requiring grooming services for their animals and experiencing financial strain; and outdoor dogs without appropriate shelter. Other frequently mentioned factors without clear patterns of association included newly acquired pets, domestic violence situations, species other than cats and dogs, housing insecurity, animal behavior issues, people needing informational resources on trap-neuter-return-monitor (TNRM), human housing-related needs, and interventions relating to legal cases (judicial orders or checks on animals returned to their owners).

### 3.2. Households

Sixty-one percent (319/519) of households included dogs, 57% (296/519) included cats, and 9% (49/519) included other species, primarily rabbits, birds, and turtles. Households frequently included more than one species of animal, including 20% (105/519) with both dogs and cats, 5% (28/519) with cats and other species, and 6% (33/519) with dogs and other species. Households where signs of object hoarding were present contained a mean of 13.00 (*SD* = 16.90) animals of any species, versus a mean of 7.00 (*SD* = 10.30) animals in households with no such signs (t(497) = 3.70, *p* < 0.001), Table 2.

**Figure 3:**
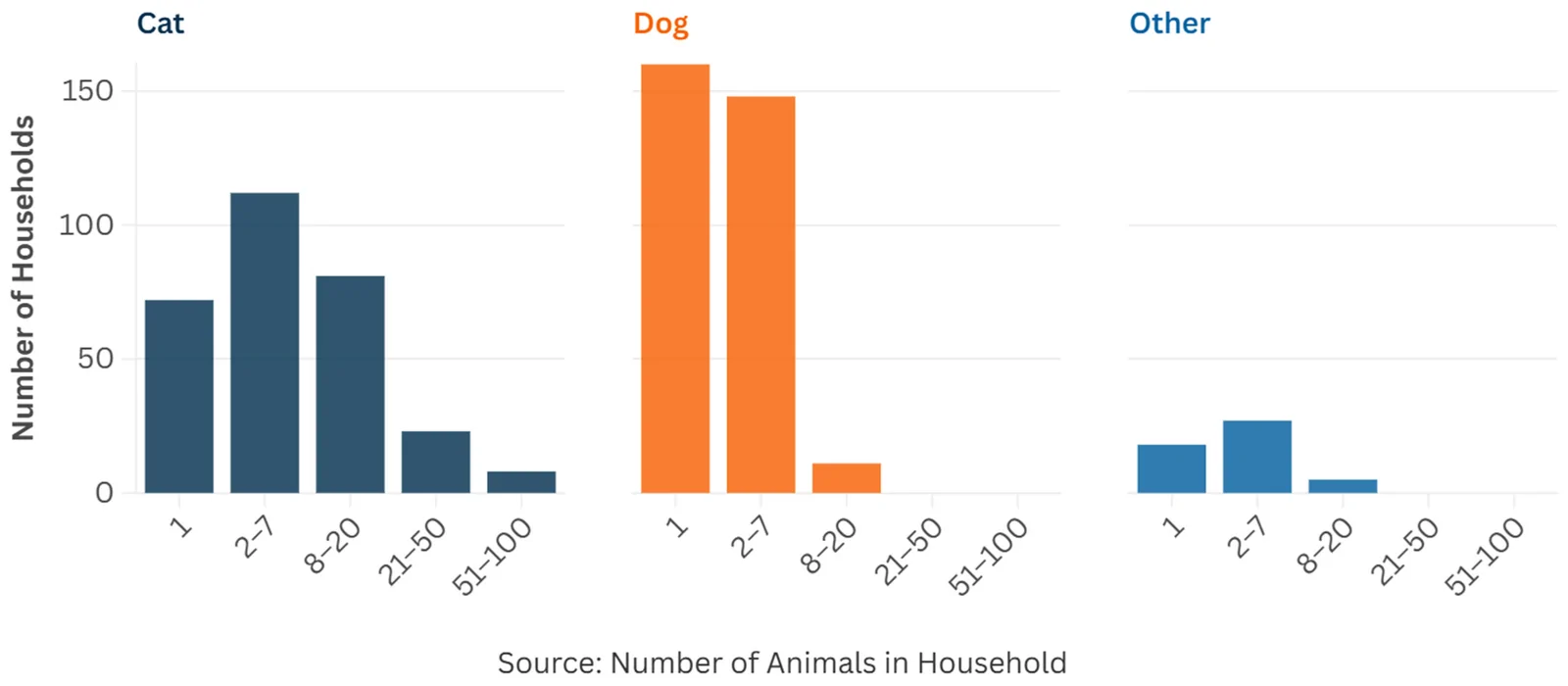
Number of animals of each species in households keeping that species.

When considering only households that owned at least one animal of a given species, cat owners had more cats than owners of other species had of their respective animals (Figure 3). On average, cat-owning households had 9.55 cats, dog-owning households had 2.29 dogs, and households with other species had 3.55 animals of those species [F(3, 515) = 29.41, *p* < 0.001]. Post hoc chi square analysis revealed significant differences between the number of cats versus dogs (*p* < 0.001) and cats versus other species (*p* = 0.03).

**Figure 4:**
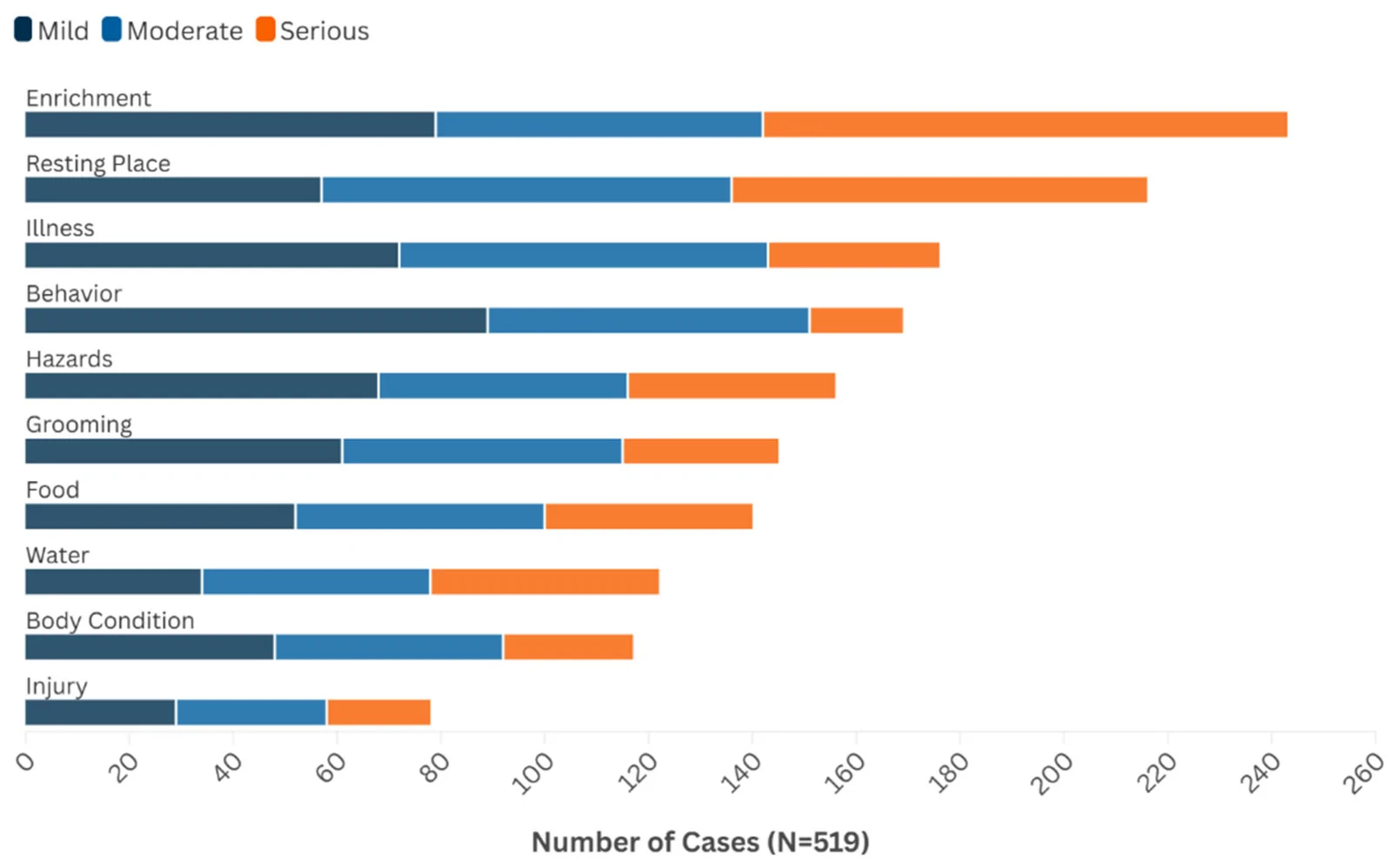
Number of cases involving each area of the Animal Needs Assessment and seriousness of welfare needs.

All ten areas of need included in the ANA were found at an appreciable rate in client households, with the number of cases involving any needs ranging from 78/519 (15%) for animal injury to 247/519 (47%) for lack of environmental enrichment (Figure 4).

Client animals had a mean of 3.08 (*SD* = 2.52) welfare needs noted (*M* = 0.87 serious needs, *SD* = 1.69). The most frequently encountered needs (of any severity) were, in descending order of frequency, lack of enrichment, lack of resting places, and illness. For all species, the most frequently encountered moderate-to-serious needs related to lack of resting places, lack of enrichment, and hazards (e.g., object hoarding, absence of running water/electricity, and lack of access to fresh air/sunlight) (Table 2).

Compared to households without cats, households with cats were more likely to present concerns relating to animal illness (Fisher’s exact *p* = 0.01,) enrichment (*p* = 0.05), and environmental hazards (*p* < 0.001). Illness-related needs were significantly associated with number of cats owned (F(3, 514) = 75.92, *p* < 0.001). Post hoc analysis using the Sidak correction revealed that fewer cats were owned in households with no illness concerns, versus moderate (Sidak, *p* < 0.001) or serious (*p* = 0.02) concerns.

### 3.3. Services

**Figure 5:**
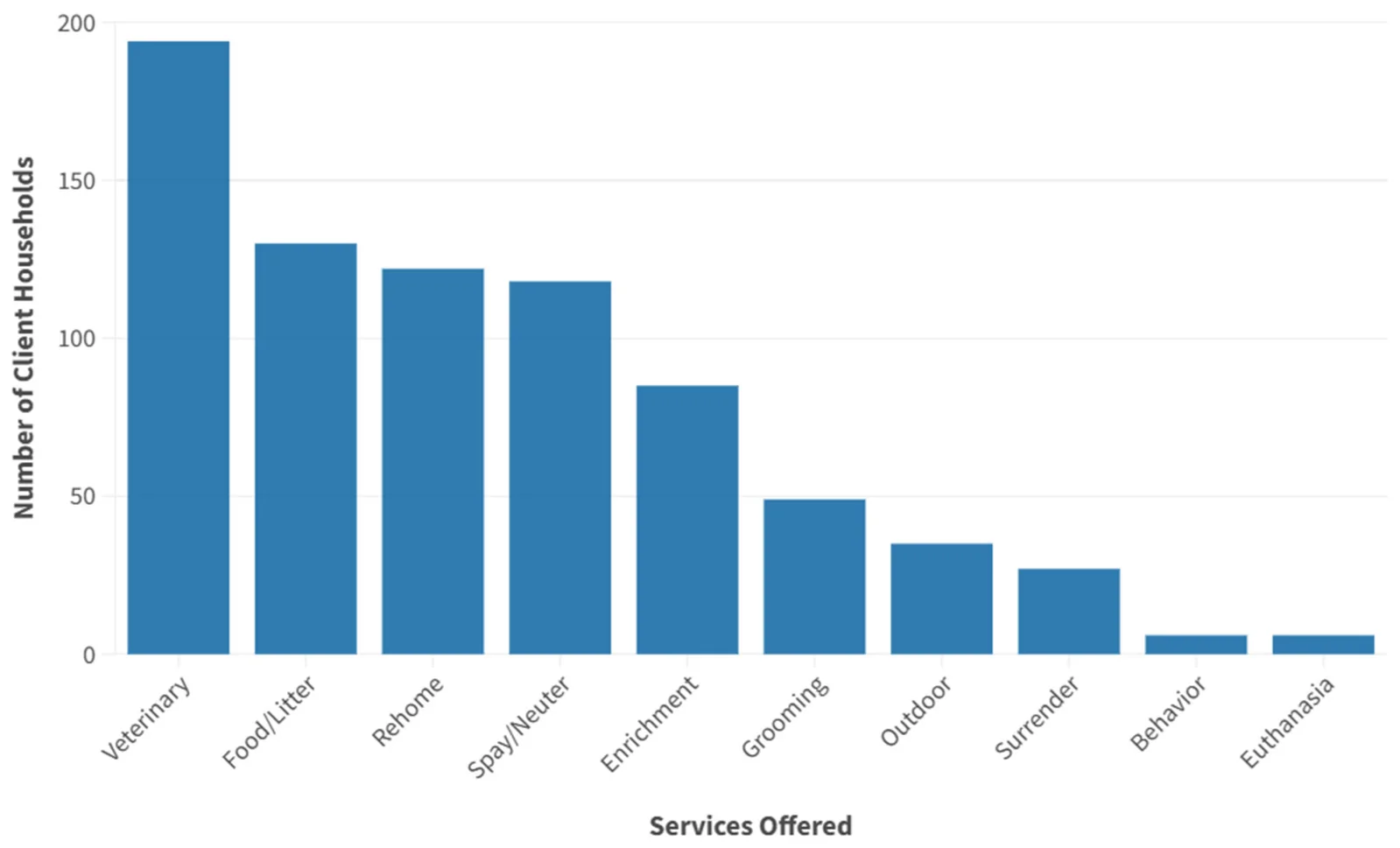
Household animal services offered based on the animal needs assessment (N = 519).

After the client’s home visit, the services to be offered were identified by CE staff based on a review of the ANA and other information recorded during the visit. Clients were offered a mean of 3.09 services (*SD* = 1.04) across 10 potential areas. In descending order of frequency, these were veterinary services (194, 37%), food/litter supplies (130, 25%), rehoming (122, 24%), spaying/neutering (118, 23%), enrichment supplies (85, 16%), grooming (49, 9%), improved outdoor housing (35, 7%), surrender (27, 5%), behavior support (6, 1%), and euthanasia (decision arrived at by pet owner with guidance from CE and veterinary professionals) (6, 1%) (Figure 5).

Eighty-nine percent (460/519) of clients were offered more than one service. To explore patterns among services offered, we conducted a principal component analysis on the correlation matrix of binary service indicator (0 = service not offered, 1 = service offered). Three components met the Kaiser criterion (eigenvalue > 1): Supplies (eigenvalue = 1.71), Population Control (1.53), and Health (1.03). These components grouped related services: Supplies included food/litter and enrichment items; Population Control included surrender and spay/neuter; Health included veterinary care and grooming.

A small proportion of cases (27/519, 5.20%) were deemed unsuitable for a collaborative approach and referred to law enforcement as suspected animal cruelty. These cases were more likely to be referred by community members who were not friends or family of the potential client (18/27, Fisher’s exact *p* < 0.001).

### 3.4. Human Service Intersections

Of the 333 clients who were asked about other social services, 62% (207) reported receiving support such as food assistance, housing aid, or home health care. During home visits, 20% (74/379) of clients requested referrals for social services. These findings reflect the overlap between human and animal welfare needs within the population served.

### 3.5. Confirming Service Delivery

Services were successfully delivered in 93% (482/519) of cases, ranging from 76% (13/17) of cases where clients were initially unwilling to participate to 94% (411/435) where clients were immediately engaged with the process. Cases were open for a mean of 41.22 ± 45.81 days (Median = 28, Range = 1–540).

A binary logistic regression was used to analyze which variables predicted successful delivery of all of the recommended services. Predictors were selected based on having a *p* < 0.05 pairwise association with successful delivery of services (as shown in Table 3). The variables “number of cats owned” and “total number of serious concerns” were omitted as being subsets of other included variables. After controlling for all other predictors, two factors were significantly associated with successful service delivery: (1) the client’s openness to receiving services when first suggested and (2) the case being referred by a source other than law enforcement (Table 4).

In 37/519 (7.13%) of cases, the client did not receive the recommended services. The most common reasons for clients not receiving services were that the owner became unavailable or unreachable, they were not cooperative, or animals were rehomed without needing ASPCA assistance.

## 4. Discussion

The ASPCA Community Engagement team developed a 10-item animal needs assessment to supplement their existing data and case narratives, help manage cases and develop a picture of the needs in New York City communities. A needs assessment approach is more common in agriculture [14,15] but is useful in any setting where the focus is on assessing and supplementing the efforts of animal caregivers.

An analysis of data from the first 519 cases using this tool demonstrated that the needs included in the assessment were commonly encountered with clients in New York City. An average of three needs were identified per household, ranging from enrichment as the most common need (243/519, 47% of cases) to injury as the least common (29/519, 6% of cases).

Overall, all items on the ANA occurred frequently and in mild, moderate and serious forms. The most common animal needs were for enrichment, and resting spaces, suggesting that while referrals frequently indicate physical issues such as veterinary care or grooming, environmental factors are also commonly noted during home visits. Enrichment for dogs and cats is more commonly researched in a laboratory or kennel setting [16]. However, many guides exist to help pet guardians understand how to make their home a more suitable habitat for their animals [17,18].

On average, each household received 3.08 services. Services were delivered in three main categories: providing supplies, population control, and health. These groupings can serve as a useful framework for categorizing interventions and guiding resource planning. Given that supply needs tended to co-occur (e.g., food, litter and enrichment items), animal welfare organizations should consider providing routine supply sets that include these items. It should be noted, however, that supplies were also sometimes offered as part of relationship-building with new clients [19], and thus did not always directly result from identified needs in these areas.

*Human–Animal Service Intersections*: The role of empathetic and client-centered communication has been recognized in other settings such as veterinary medicine [20] and animal behavior modification [21] and also appears to be important in community outreach. Client openness at first contact significantly predicted successful service delivery, to the exclusion of other variables such as number of animals owned, total number of concerns, or which services were recommended. This suggests that relational factors (trust, receptivity) may matter more than case complexity or service type [22]. Community engagement programs should emphasize the importance of training and case management procedures that assist caseworkers in building rapport with clients. This includes avoiding potential negative assumptions about categories of clients such as overwhelmed owners, who may be prematurely labeled as unwilling to receive services without full consideration of their barriers to care.

The importance of human and animal service interoperability [13,23] and One Health associations between human and companion animal wellbeing [24] was reinforced in this study. Sixty-two percent of clients who were asked about supportive services reported receiving them; social service agencies referred 20% of cases, and social service-referred cases tended to involve more serious animal needs. These findings show how social service and animal welfare organizations benefit from coordination of services, including maintaining good interagency relationships as well as cross-training, cross-reporting where applicable and required or permitted by law, and communication when interacting with the same households [25].

Social service agencies frequently assist people with some form of disability or disorder, and client needs of this type were often recorded in the text summary of the referral. However, it should be noted that the CE program does not utilize a standardized physical or mental health or disability assessment; therefore, these records likely do not reflect true prevalence of human physical or mental health issues in the population served. While it would be informative, the potential benefits of asking further about factors such as human physical and mental health must be balanced against expanding surveys to the point where they are burdensome, handling sensitive or private client information, and acting out of scope for animal professionals. However, it may be beneficial to gather more standardized data on the human elements of barriers to pet care in tandem with social service agencies.

*Access to Veterinary Care*: The most frequently provided services were veterinary, including treatments for minor illnesses or injuries, vaccines, and spay/neuter surgeries. CE staff often worked with clients to circumvent barriers preventing them from seeking these services on their own. This included subsidizing veterinary care costs, setting appointments at ASPCA clinics, and helping clients navigate challenges that would typically hinder them from attending these appointments. Client mobility issues were navigated by providing clients with rideshare services, covering costs (typically in their entirety), and setting appointments at clinics closest to client homes.

Case details taken at referral suggest that while clients who are elderly, ill or disabled face particular issues with accessing veterinary care and supplies [26], especially for dogs, most veterinary needs were at the mild or moderate levels and were met without issue. For animals with more serious veterinary issues, urgent appointments were scheduled at the ASPCA Animal Hospital, or if appropriate, these animals were referred to external veterinary clinics equipped to handle chronic illness and emergency medicine. The considerable need for veterinary services in this dataset is consistent with research indicating that access to veterinary care is a pressing concern in this community and many others [27,28], reinforcing the importance of developing and maintaining accessible veterinary services in New York City and beyond.

*Cat Population Management:* Referral case details frequently linked cat ownership with spay/neuter and other population control needs, and cat-owning clients were often described as “overwhelmed.” Difficulties with cat population control are a common community problem across the United States [29]. F Methods for addressing this in New York might include targeted outreach to multi-cat households, continued expansion of accessible spay/neuter and rehoming pathways, and collaboration with social service workers. This multi-pronged strategy may help ensure that concerns about cat numbers and welfare are identified early and addressed effectively.

Cat households also had higher illness, hazard, and enrichment concerns, reinforcing the link between cat accumulation and welfare risks. The finding that cats were more likely to show signs of illness is consistent with cats being especially prone to becoming ill in response to challenges to their welfare, such as crowding [30].

*Limitations*: The current data is specific to New York City and a population of clients referred to one agency, excluding those who decline service or result in a criminal case. As such, generalization of results should be treated cautiously, and future research should examine the complete spectrum of case pathways and outcomes to the extent possible (e.g., by combining data from community engagement programs with data from humane law enforcement efforts) to determine whether case characteristics and predictive factors for success differ. CE staff were also faced with the difficulty of losing contact with clients. This limitation in particular is not unexpected given that many clients are dealing with multiple challenges, often involving health, housing, and resources, which can affect their ability to connect via telephone calls and home visits. Also, this study commenced in January of 2021, a few months after the end of a COVID-19-related state of emergency causing many service providers to shut down (June 2020). This may have exacerbated client difficulties with finances, housing, and access to care [31].

## 5. Conclusions

The Animal Needs Assessment (ANA) proved to be a valuable tool for systematically identifying and addressing welfare concerns in New York City communities. Across 519 households, the ANA highlighted recurring issues with enrichment, resting spaces, and environmental hazards, while also capturing the scale of veterinary and population control needs. Organizing services into three categories (supplies, population control, and health) provides a clear framework for resource planning and intervention design.

A key finding was that client openness at first contact strongly predicted successful service delivery, underscoring the importance of trust-building and rapport in community engagement. The results also demonstrate the close intersection of human and animal vulnerability, reinforcing the need for collaboration between social service and animal welfare organizations.

Although the study is limited to one city and agency, the ANA offers a model that can be adapted by other organizations. Future research should expand this approach across diverse contexts, integrate data from multiple agencies, and explore strategies for sustaining long-term improvements in both animal welfare and human well-being.

## Figures and Tables

**Table 1:** The ten items of the Animal Needs Assessment. For a full description of the data entry prompts, refer to Appendix A.

1: Food	No Concerns	Mild Concerns	Moderate Concerns	Serious Concerns	Notes
2: Water	No Concerns	Mild Concerns	Moderate Concerns	Serious Concerns	Notes
3: Injury/Pain	No Concerns	Mild Concerns	Moderate Concerns	Serious Concerns	Notes
4: Illness/Disease	No Concerns	Mild Concerns	Moderate Concerns	Serious Concerns	Notes
5: Body Condition	No Concerns	Mild Concerns	Moderate Concerns	Serious Concerns	Notes
6: Haircoat/Nails	No Concerns	Mild Concerns	Moderate Concerns	Serious Concerns	Notes
7: Resting Place	No Concerns	Mild Concerns	Moderate Concerns	Serious Concerns	Notes
8: Enrichment	No Concerns	Mild Concerns	Moderate Concerns	Serious Concerns	Notes
9: Behavior	No Concerns	Mild Concerns	Moderate Concerns	Serious Concerns	Notes
10: Environmental Hazards	No Concerns	Mild Concerns	Moderate Concerns	Serious Concerns	Notes

Concern Scale—Circle which option applies and provide notes where needed. If a Moderate or Serious concern is noted for any of the factors below, the Case Lead should prioritize services to mitigate the concern for that case.

**Table 2:** Summary of demographics of cases seen by a community outreach program in New York City (N = 519).

Characteristic	Options	Number of Households
Borough	Bronx	180 (34.68%)
	Brooklyn	120 (23.12%)
	Manhattan	82 (15.80%)
	Queens	109 (21.00%)
	Staten Island	28 (5.39%)
Animals in Household	Households with dogs	319 (61.46%)
	Households with cats	296 (57.03%)
	Households with other species	49 (9.44%)
	Mean total animals/household	7.17 (*SD*: 11.30)
		Mean Number of Concerns
Concerns Noted	Mean total ANA concerns	3.08 (*SD*: 2.52)
	Mean serious concerns	0.87 (*SD*: 1.68)
	≥1 serious concern present	170 (32.76%)

**Table 3:** The number and percent of households with dogs, cats, or other species with moderate or serious needs per ANA factor.

	Dog	Cat	Other
1: Food	16.00% 51/319	17.23% 51/296	22.45% 11/49
2: Water	14.42% 46/319	16.89% 50/296	28.57% 14/49
3: Injury/Pain	9.40% 30/319	10.81% 32/296	16.33% 8/49
4: Illness/Disease	16.93% 54/319	25.34% * 75/296	30.61% 15/49
5: Body Condition	11.91% 38/319	14.86% 44/296	16.33% 8/49
6: Haircoat/Nails	17.87% 47/319	16.22% 48/296	18.37% 9/49
7: Resting Place	26.02% 83/319	34.50% 102/296	42.86% 21/49
8: Enrichment	25.71% 82/319	36.15% * 107/296	34.69% 17/49
9: Behavior	15.05% 48/319	18.92% 56/296	18.37% 9/49
10: Environmental Hazards	21.63% 69/319	28.04% * 83/296	28.57% 14/49

Asterisked values are Fisher’s Exact test with *p* < 0.05 when comparing households with that species to households without. Shaded cells represent the most common needs per species. Grayscale fill indicates the three most frequent findings for each species category.

**Table 4:** Binary logistic regression predicting successful delivery of all of the recommended services.

Log Likelihood = −115.11941			Number of Obs = 518 LR Chi2(10) = 31.18 Prob > Chi2 = 0.0001 Pseudo R2 = 0.1193			
All Services Received	Odds Ratio	Standard Error	z	*p* > [z]	[95% Conf. Interval]	
Referred by law enforcement	0.21	0.10	−3.32	<0.001	0.08	0.52
Client open to receiving services	1.67	0.33	2.56	0.01	1.13	2.46
Is this a potential criminal situation? (referred to law enforcement for further investigation)	0.20	0.14	−2.25	0.096	0.05	0.81
Service recommended: spay/neuter	0.48	0.22	−1.57	0.12	0.19	1.20
Service recommended: rehome	0.57	0.29	−1.09	0.27	0.21	1.56
Total number of concerns (any rating)	1.00	0.08	0.05	0.96	0.86	1.16
Total number of animals in household	0.98	0.01	−1.68	0.09	0.95	1.00
Total number of services recommended	0.10	0.12	−0.03	0.98	0.79	1.26
_cons	8.89	6.35	3.06	<0.001	2.19	36.05

## Data Availability

Data are not publicly available due to client confidentiality and organizational policy. Aggregated data may be provided upon reasonable request.

## Abbreviations

The following abbreviations are used in this manuscript:

ANA	Animal needs assessment
ASPCA	The American Society for the Prevention of Cruelty to Animals
CE	Community Engagement
Please note that terminology used in case records (e.g., hoarding) reflects operational language and does not imply judgment.	

## Appendix A

### CE Case Assessment Form

Purpose: This form allows CE staff to rate the animal welfare conditions of a case, advise on risk reducing measures, and assess the pet owner’s willingness to work towards implementing the recommended solutions. The assessment does not replace the staff member’s clinical judgment based on skillset and past casework experience, and it is not meant to make final decisions on the outcome of a case. The assessment is meant to lend assistance in the overall case management process, and to collect data to aid in future decision making around casework selection and strategy, resource allocation, and the likelihood of a case’s success.

- Civicore Case Number.
- Referral Source.
- Date Case Initiated.
- Pet Owner Name.
- Borough.
- # of Dogs.
- # of Cats.
- # of Other Species.
- Animal Name(s).
- AN Factor: Food—e.g., Access, Quantity/Quality, Cleanliness.
- AN Factor: Water—e.g., Access, Quantity/Quality, Cleanliness.
- AN Factor: Injury/Pain.
- AN Factor: Illness/Disease.
- AN Factor: Body Condition.
- AN Factor: Haircoat/Nails—e.g., Cleanliness, Odor, Matting/Length, etc.
- AN Factor: Resting Place—e.g., Presence of clean, low-stress area to rest and relax.
- AN Factor: Enrichment—e.g., Toys, Socialization, Outdoor Access, Exercise, etc.
- AN Factor: Behavior—e.g., Fearful/Anxious Behavior, Aggressive Behavior to People, Aggressive Behavior to Animals, etc.
- AN Factor: Environmental Hazards—e.g., Object Hoarding, Presence of Running Water/Electricity, Access to Fresh Air/Sunlight.
- Is Object Hoarding Present?
- What risk-reducing measures can be made to increase the welfare of the animal(s)?
- Is this a potential criminal situation?—If you select “Yes”—please report this case to a CE Case Manager immediately so they can make the appropriate referral to NYPD.
- Is the pet owner open to the above recommendations?
- Notes regarding pet owner’s willingness/openness to recommendations [To ask the pet owner directly]: Are there other people involved in decision-making for the pets? (e.g., family, owner, etc.).
- Are there any external factors motivating the pet owner to seek assistance with their pet(s)?—e.g., the pet owner is facing possible eviction, they are moving to another apartment, they are working with an agency like APS, a family member is providing motivation, etc.
- [To ask the pet owner directly]: We ask the next question of all of our clients. As we have relationships with many city agencies and non-profit organizations alike, we are able to offer help to our clients outside of our services. Are you currently receiving other services for yourself or your family, like from a social service agency? For example: food assistance or Medicaid.
- [To ask the pet owner directly]: Is there any other support or services that you or your family need?—If the pet owner asks for examples of services, please use “food” and/or “medical care.”
- Additional Notes.
